# Efficacy of low-level laser therapy in accelerating tooth movement, preventing relapse and managing acute pain during orthodontic treatment in humans: a systematic review

**DOI:** 10.1186/s12903-016-0242-8

**Published:** 2016-07-07

**Authors:** Mikael Sonesson, Emelie De Geer, Jaqueline Subraian, Sofia Petrén

**Affiliations:** Department of Orthodontics, Faculty of Odontology, Malmö University, Carl Gustavs väg 34, SE-205 06 Malmö, Sweden; Vasternorrland County Council, Sundsvall, Sweden; Orebro County Council, Orebro, Sweden

**Keywords:** Low-level laser therapy, Orthodontics, Pain, Relapse, Tooth movement

## Abstract

**Background:**

Recently low-level laser therapy (LLLT) has been proposed to improve orthodontic treatment. The aims of this systematic review were to investigate the scientific evidence to support applications of LLLT: (a) to accelerate tooth movement, (b) to prevent orthodontic relapse and (c) to modulate acute pain, during treatment with fixed appliances in children and young adults.

**Methods:**

To ensure a systematic literature approach, this systematic review was conducted to Goodman’s four step model. Three databases were searched (Medline, Cochrane Controlled Clinical Trials Register and Scitation), using predetermined search terms. The quality of evidence was rated according to the GRADE system.

**Results:**

The search identified 244 articles, 16 of which fulfilled the inclusion criteria: three on acceleration of tooth movement by LLLT and 13 on LLLT modulation of acute pain. No study on LLLT for prevention of relapse was identified. The selected studies reported promising results for LLLT; elevated acceleration of tooth movement and lower pain scores, than controls. With respect to method, there were wide variations in type of laser techniques.

**Conclusions:**

The quality of evidence supporting LLLT to accelerate orthodontic tooth movement is very low and low with respect to modulate acute pain. No studies met the inclusion criteria for evaluating LLLT to limit relapse. The results highlight the need for high quality research, with consistency in study design, to determine whether LLLT can enhance fixed appliance treatment in children and young adults.

## Background

It has recently been proposed that low intensity lasers, which interact with oral tissues, could improve orthodontic treatment by reducing treatment time, preventing relapse and modulating the pain of tooth movement.

Low-level laser therapy (LLLT), also known as cold laser, is a type of irradiation that does not cause a temperature rise in the tissue [1]. The mechanism of action depends on the ability of subcellular photoreceptors to respond to visible red and near-infrared wavelengths. Stimulation of these receptors influences the electron transport chain, the respiratory chain and oxidation, expressed as an increase in the cellular metabolic processes [2].

There are various potential modes of action of LLLT on the inflammatory process during orthodontic treatment, e.g. vasodilatation and induction of degranulation of mast cells, with release of proinflammatory substances to accelerate tissue healing. LLLT also increase osteoblastic and osteoclastic activity and stimulates collagen production [1].

The neuronal effect of laser therapy includes stabilization of membrane potential, inhibiting activation of the pain signal. Following laser irradiation, suppression of the pulpal response to painful stimulation has been shown in C-fibers [3]. Moreover, laser irradiation has been shown to decrease inflammatory mediators such as prostaglandin E2, known to elicit painful sensations [4].

The aim of the present study was to investigate the scientific evidence to support the application of low-level laser therapy to (a) accelerate orthodontic tooth movement, (b) prevent orthodontic relapse or (c) modulate acute pain of orthodontic treatment in children and young adults.

## Methods

To ensure a systematic approach, the literature review was conducted according to Goodman’s model [5], which consists of the following steps:

- Problem specification
- Formulation of a plan for the literature search
- Literature search and retrieval of publications
- Data extraction, interpretation of data and evidence from the literature retrieved.

The title and abstract lists were independently assessed by the four authors (MS,EDG,JS,SP). Papers of potential relevance were selected. The full-text version was analyzed and assessed according to a preset protocol by the authors, on the basis of the initial inclusion criteria. Diverging opinions were solved in consensus. The literature selection followed the PRISMA-compliant selection process [6].

### Problem specification

I. Is there evidence that LLLT is more effective than a control method in accelerating tooth movement in children and young adults during orthodontic treatment with fixed orthodontic appliances?
II. Is there evidence that LLLT is more effective than a control method in preventing relapse after orthodontic treatment in children and young adults?
III. Is there evidence that LLLT is more effective than a control method in modulating the acute pain of orthodontic treatment in children and adolescents?

The search terms used in the problem specification were defined on the basis of the United States National Library of Medicineʼs Medical Subject Headings (MeSH) prior to the literature search.

### Formulation of a plan for the literature search

Three databases were searched to identify all relevant studies: Medline (via PubMed), The Cochrane Controlled Clinical Trials Register and Scitation. The entrez date was 27/11/2015. To ensure the most comprehensive search, no MeSH terms were used, in order to avoid exclusion of recently published studies without these terms. The search strategy is presented in Table 1. The search was assisted by the staff at the Library, Malmö University, Sweden.

**Table 1:** Search strategy

	Tooth movement	Orthodontic relapse	Acute pain
Search block			
#1	Orthodontics OR Orthodontic OR Fixed Appliance	Orthodontics OR Orthodontic OR Fixed Appliance	Orthodontics OR Orthodontic OR Fixed Appliance
#2	Laser OR Low level laser therapy OR LLLT	Laser OR Low level laser therapy OR LLLT	Laser OR Low level laser therapy OR LLLT
#3	Tooth movement OR Velocity OR Rate OR Speed	Relapse OR Recurrence OR Retention	Pain OR Discomfort
#4	#1 AND #2 AND #3	#1 AND #2 AND #3	#1 AND #2 AND #3

### Literature search and retrieval of publications

Inclusion criteria were determined prior to reading the retrieved abstracts, using the population intervention control outcome method (PICO), as presented in Table 2. Sample size calculations in two studies with sufficient power [7, 8], resulted in the decision to require a minimum of twenty subjects per group.

**Table 2:** Inclusion/exclusion criteria

	Tooth movement	Orthodontic relapse	Acute pain
Inclusion criteria			
Study design	–	RCT, CCT	–
Observation period	–	Unlimited	–
Language	–	English, Scandinavian	–
Population	–	Male/female, mean age 10–30 years, sample size ≥ 20/group	–
Intervention	LLLT accelerate movement	LLLT prevent relapse	LLLT diminish acute pain
Control	–	Control or placebo	–
Outcome	Measurement in mm or per cent	–	Measurement in NRS or VAS
Exclusion criteria			
Problem specification	–	Not addressed	–
Research	–	Not original (editorial, review etc.), case series	–

Publications written in English or a Scandinavian language, addressing questions which seemed relevant to the specifications of the problem, were read in full and either included for further analysis, or excluded. The reference lists of included studies were hand searched for additional publications.

### Data extraction, interpretation and evidence from the literature

A data extraction protocol (not shown) was used to create an overview of the included studies. See Tables 3 and 4.

**Table 3:** Summary of data of the included studies on tooth movement

Study	Country	Subjects (Laser/ Placebo/ Control) Age (yrs) Gender (M/F)	Study design	Orthodontic treatment	Placebo/ Control group	Measurement	Results (laser group, LG, Control group, C)	Type of laser	Wavelength (nm)	Time per point/ Total time per treatment	Frequency of laser treatment	Power (mW)	Dose (J/cm^2^)	Time per point/ Total time per treatment	Frequency of laser treatment
Doshi-Meht [7] (2012)	India	20/20 12–23 y 8/12	Single blinded RCT (Split mouth)	Maxillary and mandibular canine retraction NiTi closed-coil spring	Placebo	Digital caliper on model	Mean increased tooth movement rate end of 3 month: LG: Maxilla; 54 %. Mandible; 58 % Mean increased tooth movement rate at complete retraction LG: Maxilla; 29 %, Mandible; 31 %	AlGaAs	800	10 s/1 min 40 s	Day 3, 7, and 14 in the first month. Thereafter on every 15th day until complete canine retraction on the experimental side, average 4.5 month	0,25	5	10 s/Unclear	Day 3, 7, and 14 in the first month. Thereafter on every 15th day until complete canine retraction on the experimental side, average 4.5 month.
Genc [9] (2013)	Turkey	20/20 17,8 y 6/14	Unblinded CCT (Split mouth)	Maxillary canine retraction NiTi closed coil spring (mini-implant)	Control	Digital calliper	Tooth movement LG; 20–40 % faster than C.	GaAlAs	808	10 s/1 min 40 s	Day 0, 3, 7, 14, 21, 28 after activation	20	0,71	10 s/1 min 40 s	Day 0, 3, 7, 14, 21, 28 after activation
Heravi [10] (2014)	Iran	20/20 22.1 y 3/17	Single blinded CCT (Split mouth)	Maxillary canine retraction	Control	Computer measurements on photos of study models	No differences between LG and C after 56 days.	GaAlAs	810	30 s/7 min 30 s	Day 4, 7, 11, 15 and 28 in the first month after Activation, Day 32, 25, 39, 43 and 56 in the second month	–	–	–	–

**Table 4:** Summary of data of the included studies on acute pain

Study	Country	Subjects (Laser/Placebo/Control) Age (yrs), Gender (M/F)	Study design	Orthodontic treatment	Placebo/control group	Pain measurement	Results (laser group, LG, laser side, LS, control, C, placebo, P)	Type of laser	Wavelength (nm)	Power (mW)	Time per point/ time per laser-treatment (second, s, minute, min)	Frequency of laser treatment (day, d, week, wk, month, mo)
Lim [20] (1995)	Singapore	39/39 21–24 y Not reported	Double blinded placebo, RCT (split mouth)	Elastomeric separators	Placebo	VAS	No difference in pain sensation	GaAsAl	830	30	15, 30, 60 s/ 1 min 15 s–5 min	One session/d during 5 d
Harazaki [19] (1997)	Japan	20/20/44 11–34 y 27/57	Single blinded RCT	Fixed appliance	Placebo and control	NRS (1–5)	Pain onset later in LG approx. 3 h	HeNe	632,8	6	30 s/ 12–24 min	One
Harazaki [11] (1998)	Japan	20/20 20,1 y 11/23	Single blinded CCT	Fixed appliance	Placebo	NRS (1–5)	LG pain reduction rate: 48.4 %	HeNe	832.8	6	30 s/ 2–5 min	One, until pain ceased
Fujiyama [12] (2008)	Japan	60/60/30 19,22 y 18/42	Single blinded CCT (split mouth)	Elastomeric separators	Control	VAS	Lower VAS separators day 4. VAS: LS 36.1, C 60.1	CO_2_	Not reported	2000	30 s/ 1 min	One
Tortamano [17] (2009)	Brazil	20/20/20 12–18 y 18/42	Double blind RCT	Fixed appliance	Placebo and control	NRS (1–5)	Lower 1th day. LG: 1.95, Placebo: 1.7, C:2.05. ended earlier LG	GaAsAl	830	30	16 s/ 32–37 min 30 s	One
Doshi-Mehta [7] (2012)	India	20/20 12–23 y 8/12	Single blinded RCT (split mouth)	Upper, lower canine retraction	Placebo	Children’s VAS	Lower VAS day 3 and 30. Day 3: LG 0.8, C 3.2. Day 30: LG 1.5, C 2.4	AlGaAs	800	0,7	30 s/Unclear	Day 0, 3, 7, 14, every 15th d in 4.5 mo.
Kim [13] (2012)	Korea	28/30/30 22,7 y 23/65	Single blinded RCT	Elastomeric separators	Placebo and control	VAS	LG lower VAS up to day 1. Overall mean VAS: LG:19.7, C:35.64	AlGaInP	635	6	30 s/ 28 min	2 times/d for 1 wk
Artés-Ribas [15] (2012)	Spain	20/20 26,4 y 6/14	Single blinded RCT (split mouth)	Elastomeric separators	Placebo	VAS	Overall mean VAS LG: 7.7, C:14.1	GaAlAs	830	100	20 s/ 3 min 20 s	One
Domínguez [14] (2013)	Colombia	60/60 24,3 y Not reported	Single blinded RCT (split mouth)	Fixed appliance	Placebo	VAS	Lower max pain on VAS. LG: 3.3, C: 6.9	GaAlAs	830	100	22 s/44 s	One
Eslamían [16] (2013)	Iran	37/37 24,97 y 12/25	Single blinded RCT (split mouth)	Elastomeric separators	Placebo	VAS	Lower VAS 6 h, 24 h, 30 h, day 3. VAS: LG:0.86, PG:1.10	AlGaAs	810	100	20 s/ 3 min 20 s	Two
Nóbrega [8] (2013)	Brazil	30/30 17,5 y 12/18	Double blinded RCT	Elastomeric separators	Placebo	VAS	LG Lower VAS VAS: LG:0.42, PG:1.88	AlGaAS	830	40,6	25–50 s/ 2 min 5 s	One
Abtahi [18] (2013)	Iran	29/29 12–22 y 24/5	Single blinded RCT (split mouth)	Elastomeric separators	Placebo	VAS	Lower VAS day 2 LG: 4.5, PG: 7.45	GaAs	904	200	7.5 s/30 s	One session/d, 5 d
Heravi [10] (2014)	Iran	20/20 22.1 y 3/17	Single blinded CCT (Split mouth)	Maxillary canine retraction	Control	–	No differences between groups after 56 days	GaAlAs	810	200	30 s/ 7 min 30 s	Day 4, 7, 11, 15, 28 1th mo.. Day 32, 25, 39, 43, 56 2nd mo.

**Table 5:** Quality evaluation protocol showing total score for studies on tooth movement

Study	Adequate selection	Single blinded	Adequate assessment of result	Adequate report ofattrition	Adequate report of side effects	No conflict of interests	Adequate study population	TOTAL
Doshi-Mehta [7] (2011)	Yes	Yes	Yes	Not reported	No	Not reported	Yes	Moderate
Genc [9] (2013)	Yes	No	Yes	Not reported	No	Not reported	Yes	Moderate
Heravi [10] (2014)	Yes	Yes	Yes	Not reported	No	Not reported	Yes	Moderate

**Table 6:** Quality of evidence that LLLT accelerates tooth movement and modulate acute pain

	Accelerating tooth movement	Modulation of acute pain
*Studies*	3	13
*Subjects*	60	333
*Study design*	RCT, CCT	RCT
*Preliminary grade of evidence*	⊕ ⊕ ⊕	⊕ ⊕ ⊕⊕
*Study quality* ^*a*^	1	1
*Inconsistency* ^*a*^	0	1
*Indirectness/Relevance* ^*a*^	1	0
*Imprecise data* ^*b*^	0	0
*Risk of publication bias* ^*a*^	0	0
*Large effect* ^*c*^	0	0
*Dose-response* ^*d*^	0	0
*Confounding factors* ^*d*^	0	0
*Overall quality of evidence*	⊕ Very low	⊕⊕ Low

^*a*^Factors that can reduce the quality of the evidence (1 or 2 levels)

^*b*^Factor that can reduce the quality of the evidence (1 level)

^*c*^Factor that can increase the quality of the evidence (1 or 2 levels)

^*d*^Factors that can increase the quality of the evidence (1 level)

**Table 7:** Quality evaluation protocol showing total score for studies on acute pain

Study	Adequate selection	Single blinded	Double blinded	Adequate assessment of result	Adequate report of attrition	Adequate report of side effects	No conflict of interests	Adequate study population	TOTAL
Lim [20] (1995)	Yes	No	Yes	Yes	Not reported	No	Not reported	No	Moderate
Harazki [19] (1997)	No	Yes	No	No	Not reported	No	Not reported	Yes	Low
Harazaki [11] (1998)	No (CCT)	Yes	No	No	Not reported	No	Not reported	Yes	Low
Fujiyama [12] (2008)	No (CCT)	Yes	No	Yes	Not reported	No	Not reported	Yes	Moderate
Tortamano [17] (2009)	Yes	No	Yes	Yes	Not reported	No	Not reported	Yes	Moderate
Doshi-Metha [7] (2012)	Yes	Yes	No	Yes	Not reported	No	Not reported	Yes	Moderate
Kim [13] (2012)	Yes	Yes	No	Yes	Not reported	No	Not reported	Yes	Moderate
Artés-Ribas [15] (2012)	Yes	Yes	No	Yes	Not reported	No	Not reported	Yes	Moderate
Dominguez [14] (2013)	Yes	Yes	No	Yes	Not reported	No	Yes	Yes	Moderate
Eslamian [16] (2013)	Yes	Yes	No	Yes	Not reported	No	Not reported	Yes	Moderate
Nóbrega [8] (2013)	Yes	No	Yes	Yes	Yes	No	Yes	Yes	High
Abtahi [18] (2013)	Yes	Yes	No	Yes	Not reported	No	Yes	No	Moderate
Heravi [10] (2014)	Yes	Yes	No	Yes	Not reported	No	Not reported	Yes	Moderate

The quality of the selected publications was assessed according to predetermined criteria for methodology and performance. The criteria of the checklist for clinical trial of *The Swedish Council on Technology Assessment in Health Care (SBU),* was modified and used (Appendix 1). Seven variables were analyzed; *adequate selection*, *blinding*, *adequate interpretation of results*, *adequate reporting of attrition, adequate reporting of side effects*, *risks for conflict of interest* and *adequate study population.* Each variable consisted of several subheadings.

The results were summarized and resulted in a *yes* or a *no* for the field. One point was awarded for each variable, except for the variable *double blinded* which was awarded two points, as double blind studies are preferred. The quality of studies awarded six to eight points was denoted as high, three to five points as moderate, and up to two points as low. Quality of evidence was rated according to Grading of Recommendations Assessment, Development and Evaluation (GRADE) guidelines, as strong, moderate, low and very low. To investigate the risk of publication bias, a search was conducted in www.controlled-trials.com and www.clinicaltrials.gov to verify the number of ongoing studies in this field. No studies were to be found.

## Results

### Accelerating tooth movement

**Fig. 1:**
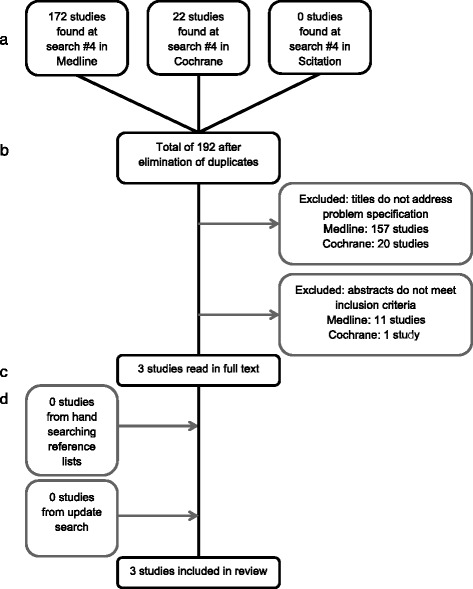
Flowchart showing studies included in LLLT and acceleration of tooth movement. **a** Identification. **b** Screening. **c** Eligibility. **d** Included

The systematic search approach, further described in Fig. 1 yielded three studies [7, 9, 10]. One trial was from India, one from Iran, and one was from Turkey. One study was designed as a randomized controlled trial (RCT) and two as controlled clinical trials (CCT), Table 3. Two of the studies reported a significant increase in velocity of tooth movement [7, 9]. One study showed an increased velocity of tooth movement of approximately 30 % in the laser treatment group compared to the control group [9]. Another study reported similar results after complete canine retraction: acceleration of 27 % in the maxilla and 31 % in the mandible [7]. Finally, one study reported no accelerated tooth movement [10], Table 3.

### Preventing relapse

**Fig. 2:**
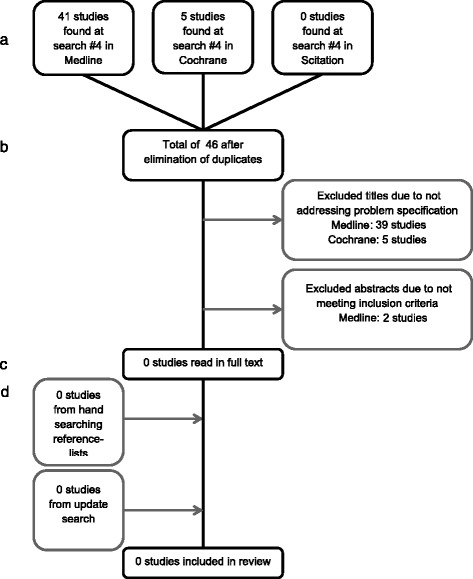
Flowchart showing studies included in LLLT and prevention of orthodontic relapse. **a** Identification. **b** Screening. **c** Eligibility. **d** Included

The systematic search approach failed to identify any relevant study that matched the inclusion criteria, Fig. 2.

### Modulating acute pain

**Fig. 3:**
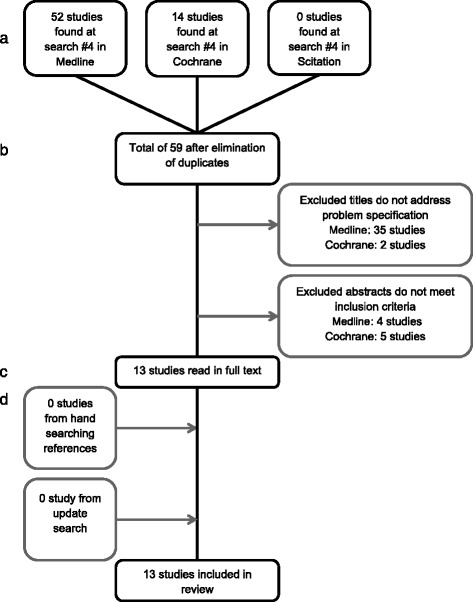
Flowchart showing studies included in LLLT and modulation of acute pain. **a** Identification. **b** Screening. **c** Eligibility. **d** Included

The systematic search approach yielded thirteen studies, Fig. 3. Two trials were from Brazil, one from Colombia, one from India, three from Iran, three from Japan, one from Korea, one from Singapore and one was from Spain. Ten studies were designed as RCT and three as CCT, Table 4. Eleven studies showed a statistically significant reduction in reported pain among the patients treated with LLLT [7, 8, 11–19]. Two studies [10, 20] found no differences in pain sensation, Table 4.

#### Quality evaluation

##### Accelerating tooth movement

After analysis, the quality of the three studies of acceleration of tooth movement by laser irradiation was rated as *moderate*, Table 5.

One study [7] included a power analysis and had single blinded subjects, but failed to report side effects. The quality of two studies [9, 10] was downgraded because the subjects were not blinded and recruitment of the participants was not described. In addition, no power analysis or control of side effects was included. Quality of evidence was rated according to GRADE guidelines as *very low* Table 6.

##### Preventing relapse

No quality analysis or quality of evidence rating according to GRADE was made.

##### Modulating acute pain

In the quality analysis, two studies [11, 19] were graded as *low* and ten studies [7, 10, 12–18, 20] as *moderate*. Only one study [8] was considered to be of *high* quality, Table 7.

All subjects in the studies on pain were blinded to their treatment; three studies used a double blind method [8, 17, 20]. Three studies included a power analysis to calculate the number of subjects needed [7, 8, 13]. However, one study [7] was on pain and treatment time and the sample size calculation was based on treatment time. Quality of evidence was rated according to GRADE guidelines as low, Table 6.

## Discussion

The present systematic review revealed that there is currently inadequate evidence to support the application of LLLT to prevent relapse. With respect to acceleration of tooth movement, the quality of evidence was very l*ow.* The quality of evidence that LLLT modulates the acute pain of orthodontic tooth movement was *low*.

The aim of the present review was to identify studies of higher quality. A limited number of studies were found and only one [8] was of high quality. However, this is a relatively recent field of research and several of the studies were published in periodicals, thus not included in the databases. Wider inclusion criteria, including studies in other languages than English or Scandinavian, might have resulted in higher numbers of studies, thus better reflecting the scientific field. However, as inclusion was limited to human studies of adequate sample size ensuring sufficient power, this review is of high clinical relevance. Using the strict guidelines of *The Swedish Council on Technology Assessment in Health Care,* the present review shows that, there is inadequate scientific evidence supporting application of LLLT to improve orthodontic treatment with respect to current indications.

The main reason for exclusion of studies was that the laser application investigated was not relevant to the scientific question specified for the present review. Other applications included e.g. measurements of casts and bonding of brackets. The inclusion criterion requiring a minimum of twenty subjects in the test group was based on sample size calculations in two studies [7, 8]. In the quality analysis, the inclusion of dental students was considered unacceptable because of the potential increase in the Hawthorne effect. However, such an assumption about the test subjects is ambiguous and could be regarded as an error in the selection method.

### Accelerating tooth movement

Several studies were excluded because they did not meet the established inclusion criteria. The main reason for exclusion was that the stated objectives did not correspond with the specifications of the research question to be addressed by the review. Four studies were excluded because of inadequate sample size [21–24]; all but one [22] reported significant acceleration of tooth movement. One study showed that irradiated teeth, compared to control teeth, moved 34 % further during the same time interval and one study showed that LLLT accelerated the initial phase of canine retraction [21, 24].

Although there are few published investigations in this field, all studies included reported similar results, LLLT accelerates tooth movement by 30 %. Doshi-Mehta et al. [7] investigated both velocity and pain. Different exposure times and output power were used to promote analgesia or biostimulation. Given the differences between power and exposure in comparison with other studies, the possibility that the analgesic regimen affected biostimulation and vice versa, cannot be disregarded.

Neither the included nor the excluded studies adequately addressed side effects of LLLT treatment. The clinical advantages and disadvantages must be considered before LLLT becomes generally available for clinical application. Rapid tooth movement increases the risk of root resorption [25], yet only one study [7] used radiographs to monitor possible radiographic changes. It is important to monitor such side effects, even though this is not a primary effect of the irradiation, but an effect of its ability to accelerate tooth movement.

Two studies [7, 9] stated that LLLT reduced orthodontic treatment time: according to the authors this could lead to further benefits for the patient as well as reduced costs. However, another study [10] showed that LLLT did not reduce treatment time. Thus to date the effects of LLLT on treatment time are unconfirmed.

### Preventing relapse

One study [26], excluded because of the small sample size (n = 14), investigated the impact of LLLT on preventing relapse, by stimulating bone remodelling after closure of a median diastema, but there was no statistically significant difference between test and control groups. As no studies were included, the question of whether LLLT can prevent relapse remains undetermined. One reason for the limited number of studies in the field might be the difficulty of study design: an extended follow-up time, preferably up to several years, is desirable. In addition, the long term side effects of using LLLT seems not to be investigated. Nevertheless, given the increasing demand from patients for long-term treatment results, this field of research is likely to warrant more attention in future.

### Modulating acute pain

Four studies [23, 27–29] on LLLT and modulation of pain during orthodontic treatment were excluded due to small sample sizes. All but one [28] of these studies reported reduced pain sensation in the LLLT group. One study [29] showed both less pain and a decrease in Prostaglandin E2 production and two studies [23, 27] showed lower pain prevalence when using LLLT.

Of the included studies [7, 8, 11–19], all but one [12] had a placebo group. The placebo groups received only light from the laser device or were irradiated with a Light Emitting Diode, LED. Since all studies scored severity of perceived pain by VAS (Visual Analogue Scale) or NRS (Numeric Rate Scale), a placebo group must be considered preferable, as it excludes any response that could interfere with perception of pain. The means used to elicit pain differed in the studies, some using elastomeric separators and others fixed orthodontic appliances. None of the studies addressed the question of whether pain elicited by an elastomeric separator is as recalcitrant as that elicited by a fixed orthodontic appliance. No correlation was discerned between the type of pain stimulus and the study results. Thus, as the method of pain induction seemed to have little impact on the result, it would be more clinically relevant to measure pain associated with fixed appliance treatment rather than separators.

In the studies on pain, the most frequently used method for measurement was VAS. In some studies [11, 17, 19] NRS was used instead. Only one study [7] used a childrenʼs VAS. None of the studies addressed the question of whether the younger participants were able to comprehend the method being applied.

Acute orthodontic pain lasts up to 7 days [30]. It is therefore of interest to note that in the study investigating the severity of pain on day 30 [7], canine retraction did not start until day 21. In one study [19] the subjects rated their pain for 14 days: this must be considered an unnecessarily long follow-up time. Moreover, five subjects in the control group experienced pain until day 14: this is difficult to explain and was not commented on by the authors.

Three studies on pain [11, 17, 19] were double blinded. Blinding was not discussed in any of the studies; although in this context, the risk of operator bias is considered to be low, double blinding would have been preferable. An inherent risk associated with the split mouth method is that the desired effect may occur on the control side as well. This issue was not addressed in any of the studies.

It is notable that none of the studies discussed side effects of laser treatment. Furthermore, no safety instructions appear to have been given to those operating the equipment. LLLT is unlikely to cause side effects in the oral environment, but should always be handled with care [31]. Although eleven out of thirteen studies reported significant modulation of acute orthodontic pain associated with application of LLLT, it was difficult to draw any conclusions because of the variation in study design. Some studies [11, 14] measured the most severe pain as the main outcome, whereas others [15, 19] focused on delayed pain or acute pain. Furthermore, the pain rating was generally low in both the placebo/control group and in the experimental group. A pain reduction of approximately one unit on the scale must unfortunately be considered to be of limited clinical relevance.

Because the studies used different definitions of pain frequency, intensity, onset and duration, these characteristics were not considered separately in the present review. The question arises as to how these aspects of pain perception might affect patient preferences. Would it be preferable to experience severe pain of short duration or mild pain over a longer period? The findings of pain modulation in the studies should be considered in the context of current knowledge about different perceptions of pain. As in all discussions of pain, the wide individual range in sensitivity needs to be taken into account.

Several studies included in this review reported quite promising results for the application of LLLT to accelerate tooth movement and modulate acute pain. In addition, two systematic review were published recently, one meta-analysis on the efficacy of LLLT for accelerating tooth movement and one on LLLT for orthodontic pain, indicating that LLLT might be a promising method to speed up the tooth movement and relieve pain during orthodontic treatment [32, 33]. However, the previous reviews had different inclusion criteria partly identifying other studies compared to the present investigation, which makes it difficult to do any comparisons of the outcome.

In this study, the laser regimens varied widely between the investigations and it is obvious that there is no consensus with respect to different lasers, frequencies and powers. Thus whether the relationship between the different laser parameters is a major determinant of effectiveness of LLLT in improving orthodontic treatment, is still open to speculation. The question of selection of laser regimen cannot be overemphasized. For instance, comparison of studies is difficult because of the confusion of concepts and terms. One example is the term *dose*, which can be referred to as J/cm^2^ or just Joules. Also, J/cm^2^ can be described as total time or per second. As it is unclear what J/cm^2^ refers to in the included studies, the term *dose* in Tables 4 and 5 is not further defined.

## Conclusions

The present systematic review reveals very *low* quality of evidence that LLLT accelerates orthodontic tooth movement and *low* quality of evidence that LLLT modulates acute orthodontic pain. No studies on LLLT to prevent orthodontic relapse met the inclusion criteria.

These findings highlight the need for consistency in study design and conformity of laser method, to determine whether LLLT is an effective method for accelerating tooth movement, preventing orthodontic relapse or modulating the acute pain of orthodontic tooth movement in children and young adults.

## Abbreviations

CCT, Controlled clinical trials; GRADE, Grading of recommendations assessment, development and evaluation; J/cm^2^, Joules per square meter; LLLT, Low-level laser therapy; MeSH, Medical Subject Headings; NRS, Numeric rate scale; PICO, Population intervention control outcome; RCT, Randomized clinical trials; SBU, The Swedish Council on Technology Assessment in Health Care; VAS, Visual analogue scale

## Appendix 1

**Table 8:** Quality template, modified version from The Swedish Council on Technology Assessment in Health Care, SBU

A1. Review of shortcomings – any systematic errors (bias)	Yes	No	Indefinite	Not applicable
A1. Selection bias				
a) Was an appropriate method for randomizing used?	–	–	–	–
b) If the study used any form of limitation within the process of randomizing (for example block, strata, minimizing), are the reasons for this adequate?	–	–	–	–
c) Was the composition of the groups adequately analogous?	–	–	–	–
d) If there was any correction for imbalances in the baseline variables, was it performed in an adequate way?	–	–	–	–
* Comments:*				
Concluding assessment:		Low:	Medium:	High:
A2. Treatment bias				
a) Were the study participants blinded?	–	–	–	–
b) Were the researchers blinded?	–	–	–	–
* Comments:*				
Concluding assessment:		Low:	Medium:	High:
A3. Assessment bias				
a) Were the observers evaluating the results blinded to the type of intervention?	–	–	–	–
b) Were the observers reviewing the outcome impartial?	–	–	–	–
c) Was the outcome adequately defined?	–	–	–	–
d) Was the outcome identified/diagnosed with a validated method of measurement?	–	–	–	–
e) Were adequate statistical methods applied to reporting the outcome?	–	–	–	–
* Comments:*				
Concluding assessment:		Low:	Medium	High:
A4. Failure bias				
a) Was the statistical handling of attrition adequate?	–	–	–	–
b) Were the reasons for attrition analysed?	–	–	–	–
* Comments:*				
Concluding assessment:		Low:	Medium:	High:
A5. Reporting bias				
a) Were side effects/complications measured in a systematic manner?	–	–	–	–
* Comments:*				
Concluding assessment:		Low:	Medium:	High:
A6. Conflicts of interest				
a) Was there a risk of conflicts of interest or of influence from a financier?	–	–	–	–
* Comments:*				
Concluding assessment:		Low:	Medium:	High:
A7. Study population				
a) Was the population from which the participants were sampled described and relevant?	–	–	–	–
b) Was the recruitment of participants acceptable?	–	–	–	–
c) Was the study population adequate?	–	–	–	–
d) Was the analysed population (ITT or PP) appropriate to the question to be addressed by the study?	–	–	–	–
* Comments:*				
Concluding assessment:		Low:	Medium:	High:
Appraisal				
A1. Selection bias	–	–	–	–
A2. Treatment bias	–	–	–	–
A3. Assessment bias	–	–	–	–
A4. Failure bias	–	–	–	–
A5. Reporting bias	–	–	–	–
A6. Conflicts of interest bias	–	–	–	–
A7. Study population	–	–	–	–
* Comments:*				
Concluding assessment of quality:		Low:	Medium:	High:

## References

[1] Chung H, Dai T, Sharma SK, Huang YY, Carroll JD, Hamblin MR (2012). The nuts and bolts of low-level laser (light) therapy. Ann Biomed Eng.

[2] Johar K (2011). Low level laser therapy. In: fundamentals of laser dentistry.

[3] Wakabayashi H, Hamba M, Matsumoto K, Tachibana H (1993). Effect of irradiation by semiconductor laser on responses evoked in trigeminal caudal neurons by tooth pulp stimulation. Lasers Surg Med.

[4] Bjordal JM, Lopes-Martins RA, Iversen VV (2006). A randomised, placebo controlled trial of low level laser therapy for activated Achilles tendinitis with microdialysis measurement of peritendinous prostaglandin E2 concentrations. Br J Sports Med.

[5] Goodman CS (1994). Report from the Swedish council on technology assessment in health care (SBU). literature searching and evidence interpretation for assessing health care practices. Int J Technol Assess Health Care.

[6] Moher D, Liberati A, Tetzlaff J, Altman DG, and The PRISMA Group (2009). Preferred reporting items for systematic reviews and meta-analyses: the PRISMA Statement. PLoS Med.

[7] Doshi-Mehta G, Bhad-Patil WA (2012). Efficacy of low-intensity laser therapy in reducing treatment time and orthodontic pain: a clinical investigation. Am J Orthod Dentofacial Orthop.

[8] Nobrega C, da Silva EM, de Macedo CR (2013). Low-level laser therapy for treatment of pain associated with orthodontic elastomeric separator placement: a placebo-controlled randomized double-blind clinical trial. Photomed Laser Surg.

[9] Genc G, Kocadereli I, Tasar F, Kilinc K, El S, Sarkarati B (2013). Effect of low-level laser therapy (LLLT) on orthodontic tooth movement. Lasers Med Sci.

[10] Heravi F, Moradi A, Ahrari F (2014). The effect of low level laser therapy on the rate of tooth movement and pain perception during canine retraction. Oral Health Dent Manag.

[11] Harazaki M, Takahashi H, Ito A, Isshiki Y (1998). Soft laser irradiation induced pain reduction in orthodontic treatment. Bull Tokyo Dent Coll.

[12] Fujiyama K, Deguchi T, Murakami T, Fujii A, Kushima K, Takano-Yamamoto T (2008). Clinical effect of CO^2^ laser in reducing pain in orthodontics. Angle Orthod.

[13] Kim WT, Bayome M, Park JB, Park JH, Baek SH, Kook YA (2013). Effect of frequent laser irradiation on orthodontic pain. A single-blind randomized clinical trial. Angle Orthod.

[14] Dominguez A, Velasquez SA (2013). Effect of low-level laser therapy on pain following activation of orthodontic final archwires: a randomized controlled clinical trial. Photomed Laser Surg.

[15] Artes-Ribas M, Arnabat-Dominguez J, Puigdollers A (2013). Analgesic effect of a low-level laser therapy (830 nm) in early orthodontic treatment. Lasers Med Sci.

[16] Eslamian L, Borzabadi-Farahani A, Hassanzadeh-Azhiri A, Badiee MR, Fekrazad R (2013). The effect of 810-nm low-level laser therapy on pain caused by orthodontic elastomeric separators. Lasers Med Sci.

[17] Tortamano A, Lenzi DC, Haddad AC, Bottino MC, Dominguez GC, Vigorito JW (2009). Low-level laser therapy for pain caused by placement of the first orthodontic archwire: a randomized clinical trial. Am J Orthod Dentofacial Orthop.

[18] Abtahi SM, Mousavi SA, Shafaee H, Tanbakuchi B (2013). Effect of low-level laser therapy on dental pain induced by separator force in orthodontic treatment. J Dent Res.

[19] Harazaki M, Isshiki Y (1997). Soft laser irradiation effects on pain reduction in orthodontic treatment. Bull Tokyo Dent Coll.

[20] Lim HM, Lew KK, Tay DK (1995). A clinical investigation of the efficacy of low level laser therapy in reducing orthodontic postadjustment pain. Am J Orthod Dentofacial Orthop.

[21] Cruz DR, Kohara EK, Ribeiro MS, Wetter NU (2004). Effects of low-intensity laser therapy on the orthodontic movement velocity of human teeth: a preliminary study. Lasers Surg Med.

[22] Limpanichkul W, Godfrey K, Srisuk N, Rattanayatikul C (2006). Effects of low-level laser therapy on the rate of orthodontic tooth movement. Orthod Craniofac Res.

[23] Youssef M, Ashkar S, Hamade E, Gutknecht N, Lampert F, Mir M (2008). The effect of low-level laser therapy during orthodontic movement: a preliminary study. Lasers Med Sci.

[24] Sousa MV, Scanavini MA, Sannomiya EK, Velasco LG, Angelieri F (2011). Influence of low-level laser on the speed of orthodontic movement. Photomed Laser Surg.

[25] Proffit WR, Proffit WR, Fields HW, Sarver DM (2013). The biological basis of orthodontic therapy. Contemporary Orthodontics.

[26] Zahra SE, Elkasi AA, Eldinb MS, Vandevska-Radunovicc V (2009). The effect of low level laser therapy (LLLT) on bone remodelling after median diastema closure: A one year and half follow-up study. Orthod Waves.

[27] Turhani D, Scheriau M, Kapral D, Benesch T, Jonke E, Bantleon HP (2009). Pain relief by single low-level laser irradiation in orthodontic patients undergoing fixed appliance therapy. Am J Orthod Dentofacial Orthop.

[28] Esper MA, Nicolau RA, Arisawa EA (2011). The effect of two phototherapy protocols on pain control in orthodontic procedure—a preliminary clinical study. Lasers Med Sci.

[29] Bicakci AA, Kocoglu-Altan B, Toker H, Mutaf I, Sumer Z (2012). Efficiency of low-level laser therapy in reducing pain induced by orthodontic forces. Photomed Laser Surg.

[30] Krishnan V (2007). Orthodontic pain: from causes to management—a review. Eur J Orthod.

[31] Coluzzi DJ, Convissar RA, Convissar RA (2011). Laser fundamentals. Principles and practice of laser dentistry.

[32] Ge MK, He WL, Chen J, Wen C, Yin X, Hu ZA, Liu ZP, Zou SJ (2015). Efficacy of low-level laser therapy for accelerating tooth movement during orthodontic treatment: a systematic review and meta-analysis. Lasers Med Sci.

[33] Li FJ, Zhang JY, Zeng XT, Guo Y (2015). Low-level laser therapy for orthodontic pain: a systematic review. Lasers Med Sci.

